# Effectiveness of pharmacological interventions for Sjogren syndrome - A systematic review

**DOI:** 10.4317/jced.59891

**Published:** 2023-01-01

**Authors:** Ruchika Choudhary, Sujatha S. Reddy, Rakesh Nagaraju, Ravleen Nagi, Pooja Rathore, Ritu Sen

**Affiliations:** 1 Assistant Professor, Jaipur Dental College and Hospital, Maharaja Vinayak Global University, Jaipur, Rajasthan; 2 Professor, Department of Oral Medicine and Radiology, Faculty of Dental Sciences, Ramaiah University of Applied Sciences, Bangalore, Karnataka, India; 3 Professor and Head, Department of Oral Medicine and Radiology, Faculty of Dental Sciences, Ramaiah University of Applied Sciences, Bangalore, Karnataka, India; 4 Reader, Department of Oral Medicine and Radiology, Saveetha Dental College, Velappanchavadi, Chennai, Tamil Nadu, India; 5 Post Graduate Student, Department of Oral Medicine and Radiology, Faculty of Dental Sciences, Ramaiah University of Applied Sciences, Bangalore, Karnataka, India

## Abstract

**Background:**

Sjogren’s Syndrome (SS) is characterized by xeropthalmia and/or xerostomia. Treating the associated salivary gland hypofunction has been challenging to the clinicians. A variety of topical and systemic therapies have been tried to restore/stimulate the gland function or replace saliva reducing the symptoms of xerostomia and to avoid the problems of diminished salivary flow.

**Material and Methods:**

Four search engines (PUBMED/Medline, EMBASE, Google Scholar and The Cochrane) were used in conducting a systematic review using the terms “Sjogren’s syndrome” with the combination of other terms. To define these study acceptability criteria, we used PICO model (Population, Intervention, Control and Outcome) and study design technique.

**Results:**

Out of 47 articles initially screened, 28 studies met our selection criteria. Included studies showed positive results with interventions such as pilocarpine, rituximab, and interferon-alpha (IFN-α) for enhancing salivary flow and lacrimal secretion in SS condition. One study showed promising results for combination of prednisone and hydroxychloroquine in SS, however dose of prednisone is recommended to be tapered. Another study demonstrated comparable effects of dehydroepiandrosterone and the placebo in alleviation of dry mouth symptoms (*p*=0.006). Therapeutic effects have been reported with LASER therapy.

**Conclusions:**

Pilocarpine was found to be highly beneficial whereas, rituximab and IFN-α were moderately effective in the reduction of hyposalivation in SS patient. Adverse events were common. Use of any alternative modalities for the management cannot be supported based on the current evidence; this demands more studies in future to be conducted staking into account adverse effects which might occur particularly with the pharmacological therapies.

** Key words:**Sjogren’s Syndrome, Xerostomia, Hyposalivation, Pilocarpine, Rituximab, Sialagogue.

## Introduction

Sjogren’s Syndrome (SS) is considered autoimmune, chronic inflammatory condition, which mainly affects the exocrine glands with sicca symptoms following, such as xerostomia (dry mouth), xerophthalmia (dry eyes) and parotid gland enlargement. It is often times referred to as an autoimmune exocrinopathy’ (Fox and Speight, 1996). Approximately, 0.5-1% of the population, involves middle-aged women more frequently as compared to men, with ratio of 9:1 (1). The condition usually arises between fourth to sixth decade of life, although it can appear at any age. Various factors found to be associated with the pathogenesis of disease include immunologic, inflammatory, genetic, epigenetic, environmental, hormonal, and infectious agents (Mavragani & Moutsopoulos, 2010). It can be primary sjogren’s syndrome (pSS) which frequently occurs when there is no underlying cause for rheumatic disorder or secondary sjogren’s syndrome (sSS) that is reported to be related with another rheumatic disease, such as systemic lupus erythematosus, scleroderma, rheumatoid arthritis (RA), dermatomyositis, or primary biliary cirrhosis.

Circulating autoantibodies (anti-Ro, anti-La, ANA, etc.) and lymphocytic infiltrates in exocrine glands are the characteristic autoimmune features of the condition. Both findings are considered in the diagnostic criteria (Vitali *et al*., 2002). In relation to the same context, the inflammatory cells play a major role in the pathogenesis, attacking the epithelial cells (Manoussakis & Kapsogeorgou, 2010). As suggested by an important body of evidence, other factors promote the loss of epithelial cell homeostasis, occurring in the pre-autoimmune phase or independent of inflammatory cells (Delaleu *et al*., 2011, Perez *et al*., 2000, Ewert *et al*., 2010). Many classification criteria have been suggested for pSS (2,3). The American-European Consensus Group (AECG) proposed a classification in 2002, which is widely accepted now-a-days. Other classifications that have been accepted as a diagnosis criteria are the ones proposed by the Sjogren’s International Collaborative Clinical Allia and American College of Rheumatology (4).

The management of hypofunction of salivary glands associated to SS has been a challenging aspect for the clinicians (Montgomery-Cranny *et al*., 2014). Topical and systemic interventions have been used in several individuals with SS to attempt restoring/stimulating the functions of salivary glands or replacing saliva, so to lessen the distressing symptoms of xerostomia and preventing complications of decreased or deficient salivation (Saraux *et al*., 2016). Other alternatives for stimulating residual salivary function includes topical sialogogues such as sugar-free chewing gum and lozenges (Glore *et al*., 2009), and para-sympathomimetics drugs like cevimeline (Petrone *et al*., 2002) and pilocarpine (Vivino *et al*., 1999). In the conditions where the gland is irreversibly compromised, varied range of saliva substitutes including sprays and gels can be used (Saraux *et al*., 2016). Traditional disease-modifying anti-rheumatic drugs (DMARDs) are known to have little to no effect on salivation (van Nimwegen *et al*., 2016); however, more recent B cell-targeted agents have been recommended (Rituximab, an anti-CD20 agent) and/or suggested to represent the positive therapeutic/management strategy, including agents targeting B-cell homeostasis cytokines (e.g., IL-6 and BAFF) (Vivino *et al*., 2016, Cornec *et al*., 2013). Non-pharmacological therapies such as acupuncture (Cafaro *et al*., 2014) and salivary neuro-electrostimulation (Fedele *et al*., 2008) have also been used to improve saliva production and alleviate dry mouth symptoms. Overall, there is a scarcity of solid evidence to inform and guide the clinicians about the effectiveness of various therapies that can be utilized in the treatment of hypo-functioning of salivary glands and symptoms of xerostomia associated with SS.

The existing systemic reviews published in the literature have predominantly focused on the SS and have included the studies recruiting the investigations of SS and different treatment modalities in different etiological factors.

As a result, treatment decisions in everyday practice are likely to be relied on a combination of personal experience, expert opinion, and low-quality evidence from published studies. As a result, we conducted this multi-therapy systematic review in order to assess and estimate the efficacy of available treatment choices in individuals with SS.

## Material and Methods

-Focused Questions

Based on the PRISMA guidelines and Preferred Reporting Items for Systematic Reviews, a focused question was constructed. The addressed focused questions were.

1. Effective management of oral manifestation of Sjogren’s syndrome?

2. What are the most commonly prescribed treatment regimens for SS?

3. Adverse effect during or post-treatment?

-Study Inclusion Criteria/ Eligibility Criteria

Study inclusion criteria were (i) design: randomized controlled trials (RCT), original investigations, clinical studies: (ii) population: above 18 years diagnosed with SS (pSS or sSS) (iii) intervention: investigation and treatments designed to treat oral manifestation of SS; (iv) control group: placebo, another active intervention, no treatment or in conjunction of the aforementioned. Any route, formulation or dose can be used for administering the interventions. The studies that have been included should contain sufficient, clear information on the effect of the experimental treatment upon the clinical outcomes and written solely articles in English. Review papers, experimental research, letter to the editors, and unpublished works were not taken into account.

-Literature search/ Search Strategy

**Figure 1:**
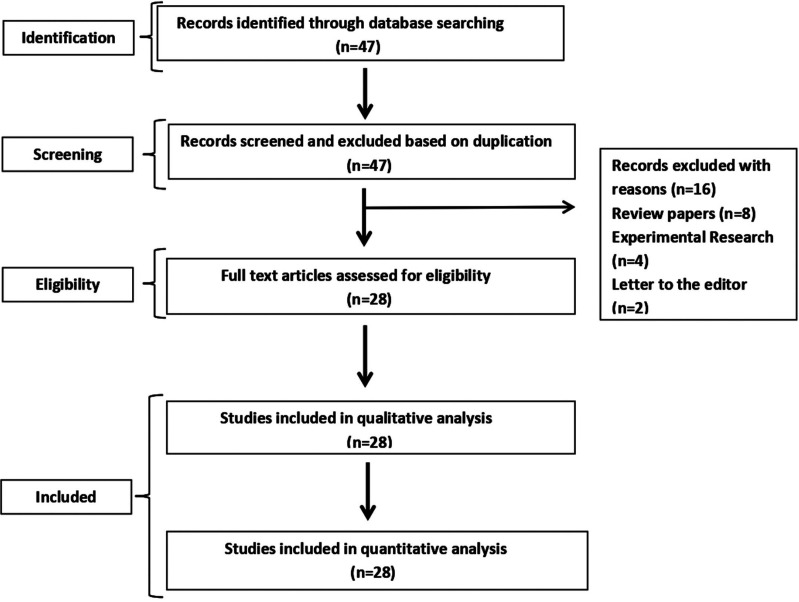
Study Selection process.

For the identification of studies included for this review, we developed detailed search strategies and each database (PUBMED/Medline, Google Scholar, The Cochrane Central Register of Controlled Trials and EMBASE) were searched from 2000 to 2021. The following terms were used in the search of all trials registers and databases: xerostomia, hyposalivation, Sjogren’s syndrome, treatment, sialagogue, saliva substitute etc. Two authors independently reviewed and double-checked the title and abstracts of articles. Using the abstract and title eligibility criteria, the whole texts of eligible articles were reviewed and independently assessed. The authors then discussed and agreed on reference lists for original and review research that they considered was relevant (Fig. 1). In the initial search 47 articles were screened. Out of them, 28 studies were included depending on the eligibility criteria and data was extracted. For summarizing the important data, the current study was designed. This systematic review strictly adheres to the PRISMA statement (Moher *et al*., 2009).

## Results

The study selection process is represented in Figure 1, which included 28 studies in a systematic review. The following classification criteria were used in the diagnosis of patients with SS condition who were selected for this study: Fox’s classification criteria (Fox *et al*., 1986), Copenhagen criteria for Sjogren’s syndrome (Manthorpe *et al*., 1986), the preliminary criteria for the classification of Sjogren’s syndrome (Vitali *et al*., 1993) and the American European Consensus Group (AECG) Sjogren’s syndrome classification criteria (Revised Europe Community Study Group) (Vitali, 2002).

Interventions included topical saliva substitutes (1 studies), topical saliva stimulants (3 studies), systemic cholinergic agonists (4 studies), LASER therapy (2 studies), biologic response modifier biological agents (6 studies), disease modifying anti-rheumatic drugs (1 studies) and Dehydroepiandrosterone (1 studies), combination of systemic cholinergic agonist and anti-rheumatic drugs (1 studies), Corticosteroids (7 studies.

## Discussion

● Topical salivary stimulants vs. Alternative salivary stimulants or placebo (lozenge)

Casey Means *et al*. (2017) (5) reported a case of a healthy male patient aged 10 years with the history of bilateral juvenile recurrent parotitis and bilateral floor-of-mouth ranulas. The patient’s medical history was important for the subjective complaints of dry eyes and history of dental caries. The patient and his family denied a history of trauma in relation to floor of the mouth, before the onset of swelling on both sides. He continued to have very frequent episodes of bilateral parotid gland swelling, treated with massage and sialagogues.

● Cholinergic agonists (Systemic) (cevimeline and pilocarpine) vs. placebo or saliva substitutes M. Cifuents *et al*. (2018) (6) reported double-blinded, randomized, controlled study in that 72 patients (69 women and 3 men) with SS were randomly assigned to receive 10 drops of pilocarpine (5mg) or 10 drops of artificial saliva, orally, three times/day for 12 weeks. They concluded that pilocarpine is more effective than artificial saliva for enhancing salivary and lacrimal secretion in patients with SS. Sialorrhea and nausea were two most common side effects reported.

Athena S. *et al*., (2004), (7) reported a trial that was placebo-controlled in 256 patients evaluating the effectiveness and safety of pilocarpine (orally) (20 mg -30 mg/daily) to relieve the symptoms of associated with SS. Symptoms and salivary flow changes were recorded over a 12-week period. Compared to placebo, the patients who received pilocarpine have shown significantly increased secretion after the first dose and through-out the study (*P* ≤ 0.0001). Throughout the study, treated patients reported a positive improvement in the overall evaluation of patients with xerostomia/dry mouth (*P* 0.0001) and 5 out of 7 separate oral symptoms were alleviated (*P* ≤ 0.02). At 12 weeks (5-7.5 mg dose), both pilocarpine groups were of the overall dry ice core, whereas 3 of the 8 ocular symptoms showed a slight difference at 6 weeks (5 mg dose). Significant improvement was shown (*P* ≤ 0.0001) and 6 of the 8 associated symptoms (*P* ≤ 0.04) were alleviated. At both the doses, the drug was reported to be well tolerated. The most commonly noted pilocarpine-related side effects were urinary frequency, sweating, flushing and chills. The authors found a significant improvement in xerostomia/dry mouth symptoms at 20 mg/daily and also reduced ocular symptoms, including a reduced need for artificial tears, after increasing the dose to 30 mg / day was noted.

Rose S. Fife *et al*. (2002) (8) For 6 weeks, researchers compared cevimeline 30 mg and 60 mg to placebo and found that there was no significant differentiation within the number of individuals who experienced a short-term (60 minutes) improvement in overall xerostomia score (3-point Likert). No changes were noted in VAS score (xerostomia) between the cevimeline and placebo groups (on a scale). However, the short-term changes in flow of saliva were significantly improved in the cevimeline group than reported in patients received placebo [0.194 vs. 0.015 mL / min (mean change). P0.05], clinical significance is unknown, but it shows a large effect size. In this study, risk of attribution obfuscation was considered unknown.

Dianne Petrone *et al*. (2007) (9) reported a study where patients had been randomly randomized to take either placebo, 15mg of cevimeline 3 times/daily, or 30mg of cevimeline 3 times/daily in a 12-week research. The authors concluded that treating patients of SS with cevimeline (dosage 30 mg 3times/day) resulted in remarkable improvements in tear and salivary flow, in addition, relieving of subjective feelings of dry eyes, dry mouth and overall dryness. Both the dosages were well tolerated by the patients, and the 15mg dosage reduced some symptoms.

Seong - Min Kweon *et al*. (2018) (10) presented a rare case of amyloidosis, localized to the lacrimal gland, with SS in 45 years old women, her diagnosis was pSS based on the presented symptoms and results of the Schirmer test, serological testing, and biopsy of minor salivary gland (SG). *Pi*locarpine (10mg/d) and hydroxychloroquine (200mg/d) were initiated for managing patients with SS. The symptoms of dry mouth and eyes did not worsen after 6 months of initial diagnosis and no lumps suggestive of localized amyloidosis were found.

● Disease modifying anti-rheumatic drugs (DMARD) vs. placebo (cyclosporine A, hydroxychloroquine, azathioprine, rebamipide)

Kristin Houghton *et al*. (2005) (11) published a case series which included 7 children diagnosed with pSS reported at British Columbia’s Children’s Hospital (BCCH) and were reviewed for pediatric and AECG criteria for pSS. Most of the patients were treated with hydroxychloroquine and it was found to be helpful in resolution of xerostomia and dysphagia with no additional anaphylactic episodes or new symptoms.

Alternative interventions vs. Alternative interventions or placebo (dehydroepiandrosterone)(DHEA)

A. Hartkamp *et al*. (2007) (12) reported a RCT (double blinded) which was placebo-controlled, 60 female patients having pSS received placebo or 200 mg oral DHEA. The outcome measures that were primarily noticed were depressive mood, mental well-being, general fatigue and physical functioning. Pain, sicca complaints, and disease activity indicators were also assessed. Patients were evaluated before starting the treatment and at 3rd, 6th, and 12th month after commencement of the medication, and at 6th month after stopping the medication. General weariness (*p* = 0.001), mental well-being (*p* = 0.04), and sad mood (*p* = 0.008) were all improved in both placebo-treated and DHEA groups. Physical performance remained unchanged (*p* = 0.44). Complaints of xerostomia reduced in both groups (*p* = 0.006), complaints of dry eyes were found to be improved with the placebo group (*p* = 0.01) and ESR decreased in the DHEA group (*p* = 0.02), according to the secondary outcome variables. Although both placebo and DHEA alleviated well-being and fatigue in female patients with pSS, this study did not demonstrate a stronger impact of DHEA when compared with placebo. This could point to need of potential cognitive behavioral therapies.

Rituximab

Z Touma *et al*. (2005) (13) reported a case report of 77-year-old women diagnosed with SS. With persistent enlargement of the parotid gland, sicca symptoms, active renal disease, and frequent recurrences of purpura and epigastric discomfort, the patient was managed with hydroxychloroquine, azathioprine, methotrexate, and cyclophosphamide (1 g/month for 6 months following with 1 g/3 months continued to be for 1 year). Prednisone was given on a daily basis but the dose was never tapered below 10 mg/day. Patient received 3 doses of infliximab 5 mg/kg due to the persistence of her symptoms, which resulted in a partial relief in her purpura and mouth dryness, but after the third treatment, patient developed pneumonia with sepsis, necessitating admission to the critical care unit. The authors decided to treat the patient with rituximab, an anti-CD20 antibody (375 mg/m2/week for 4 weeks) because her condition was refractory. Significant subjective improvement in dry eyes, parotidomegaly, dry mouth, disappearance of the purpura and decrease in the proteinuria were reported by the patient (Table 1, Table 1 cont-5). The patient was kept on prednisone 5mg/daily and remained in remission for 6 months following the last dose of rituximab. No side-effects were noted. Z touma *et al*. (13).conclude that favorable clinical efficacy and tolerability was demonstrated by rituximab and it also gave a promising alternative in specific patients with SS who were unmanageable with conventional treatment.

**Table 1:**
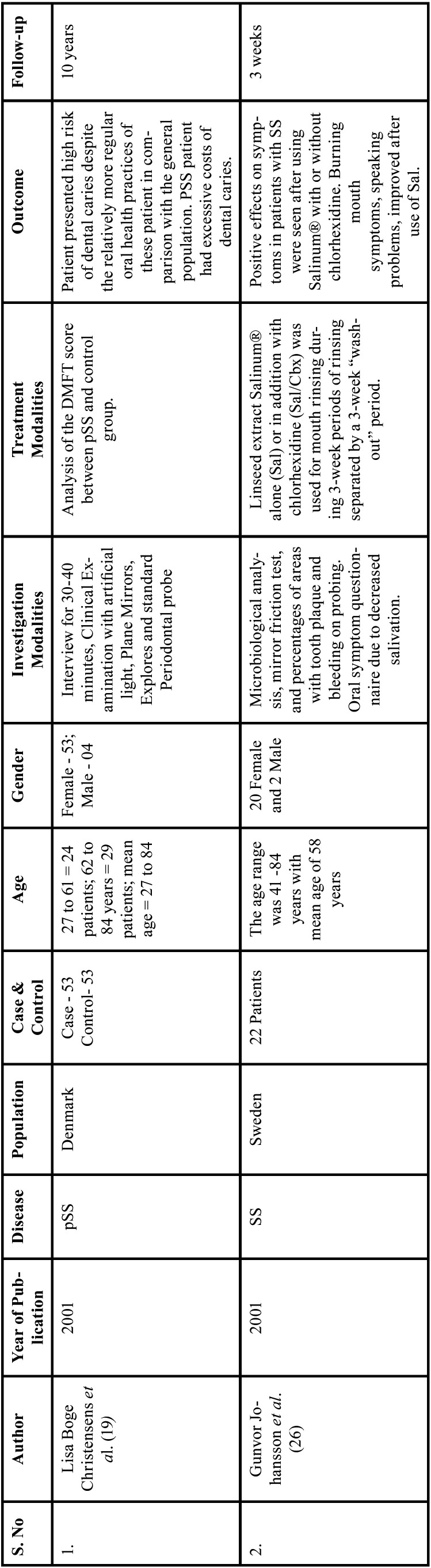
Charaterstics of included studies based on Population, Intervention, Comparison, and Outcome(PICO) model.

**Table 1 cont.**
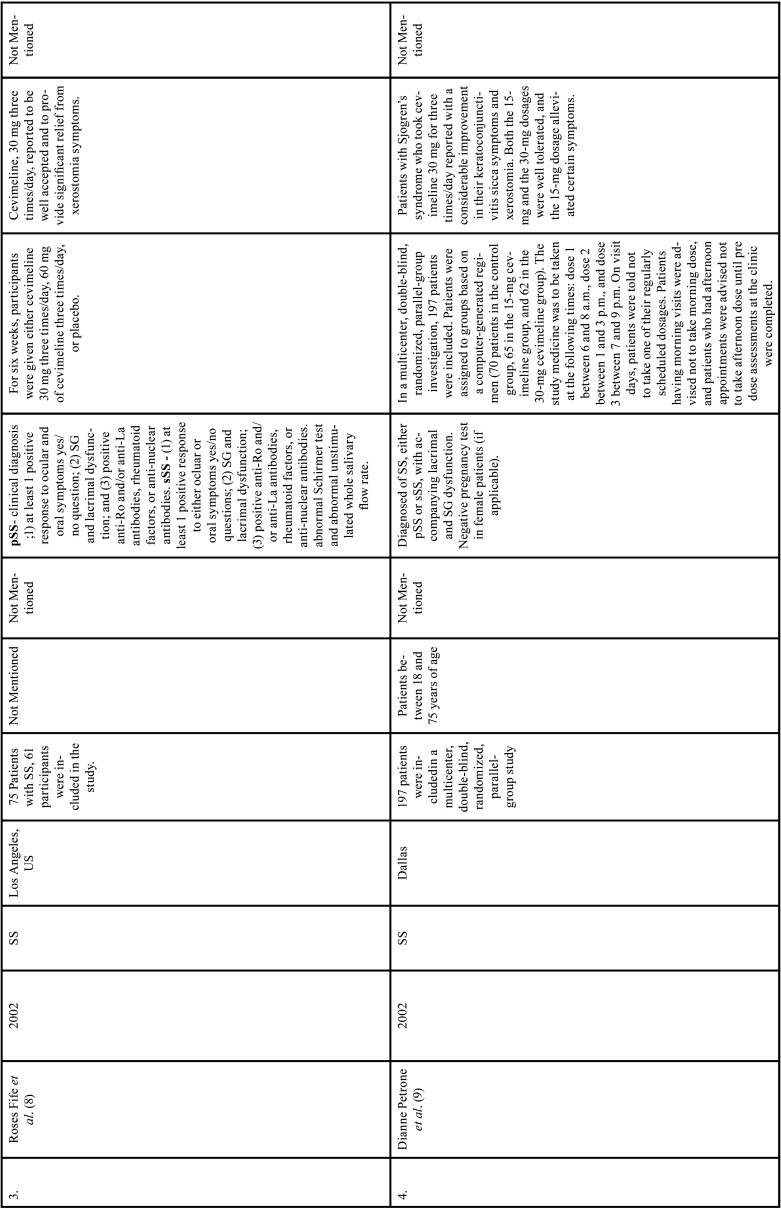
Charaterstics of included studies based on Population, Intervention, Comparison, and Outcome(PICO) model.

**Table 1 cont.-1:**
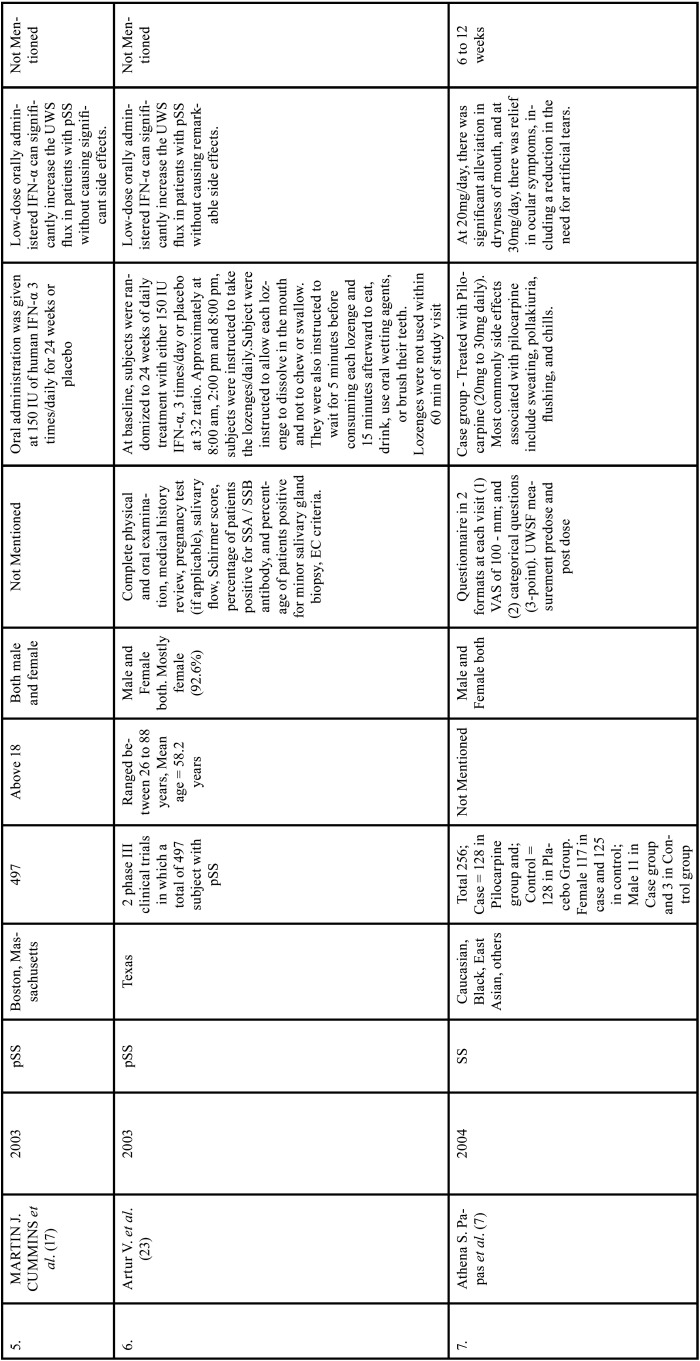
Charaterstics of included studies based on Population, Intervention, Comparison, and Outcome(PICO) model.

**Table 1 cont.-2:**
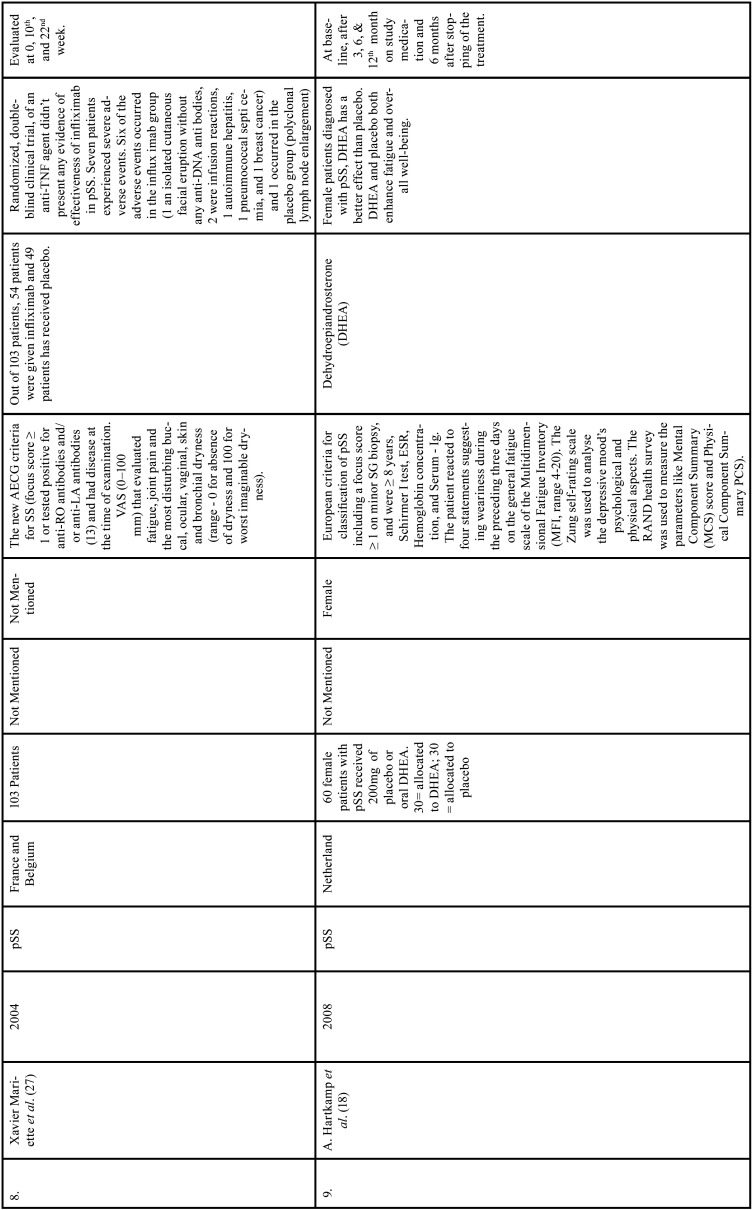
Charaterstics of included studies based on Population, Intervention, Comparison, and Outcome(PICO) model.

**Table 1 cont.-3:**
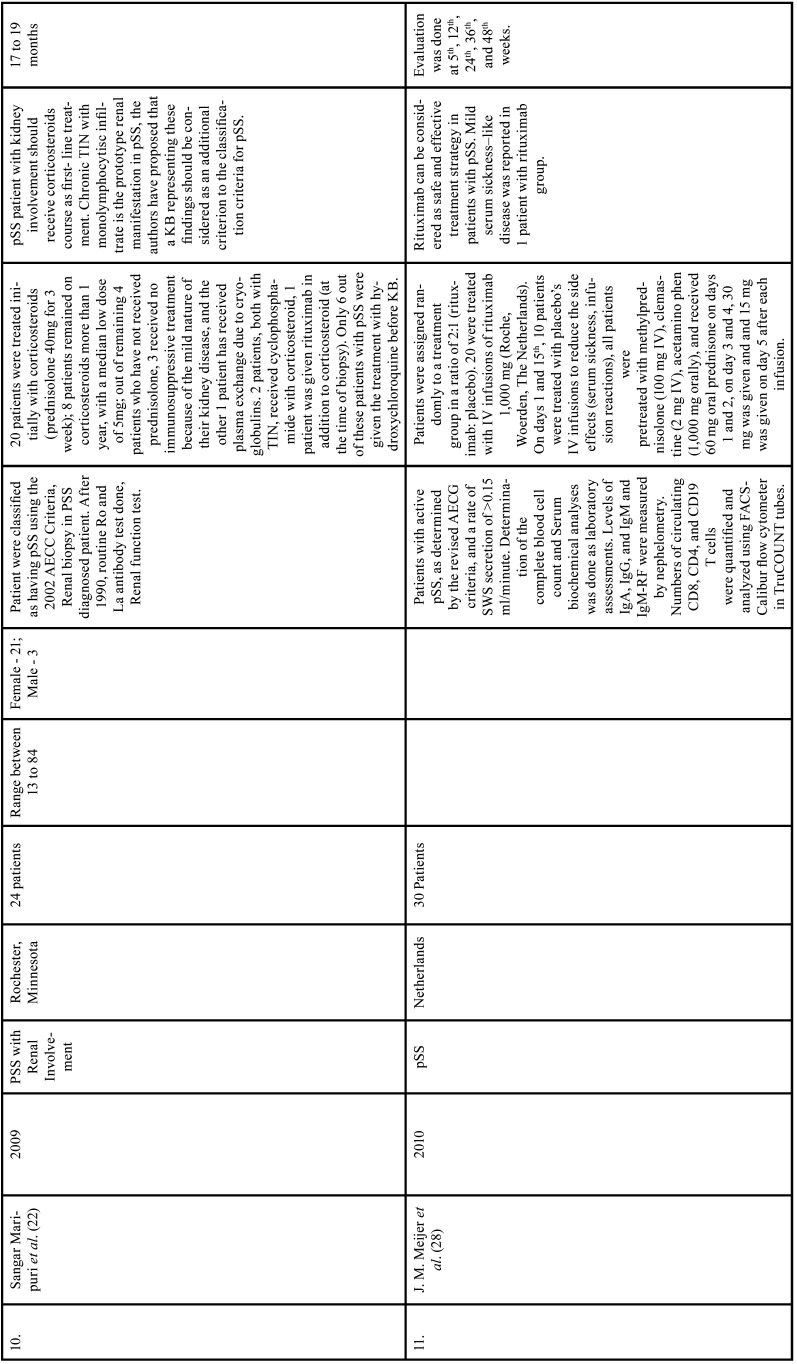
Charaterstics of included studies based on Population, Intervention, Comparison, and Outcome(PICO) model.

**Table 1 cont.-4:**
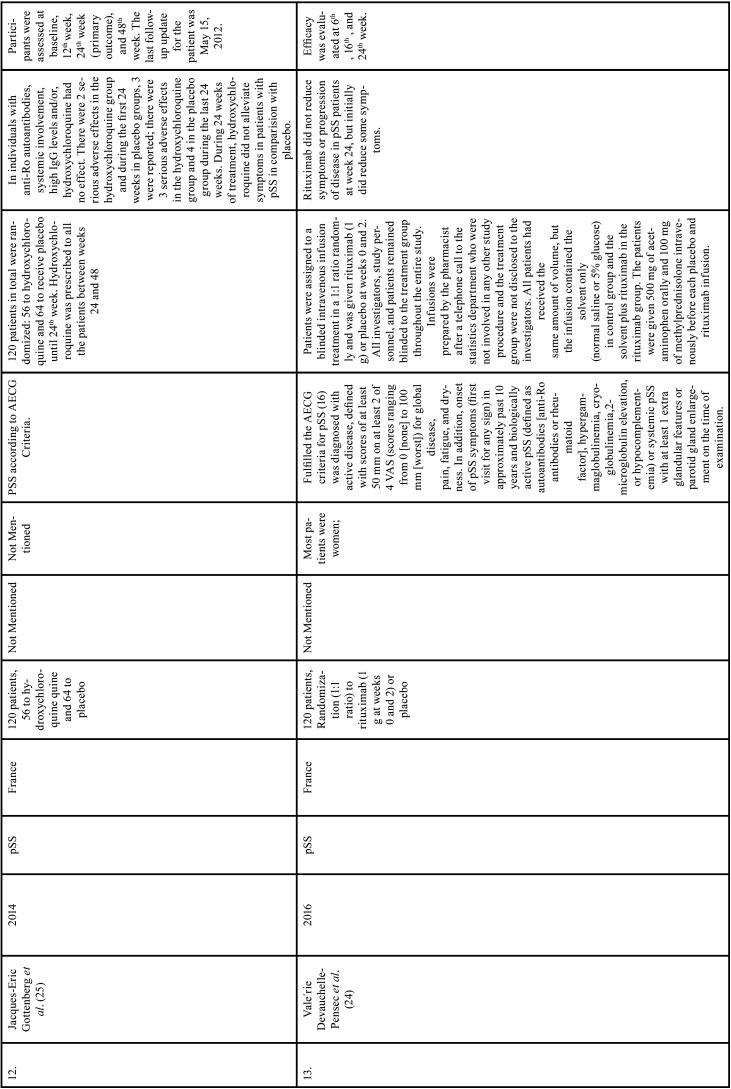
Charaterstics of included studies based on Population, Intervention, Comparison, and Outcome(PICO) model.

**Table 1 cont.-5:**
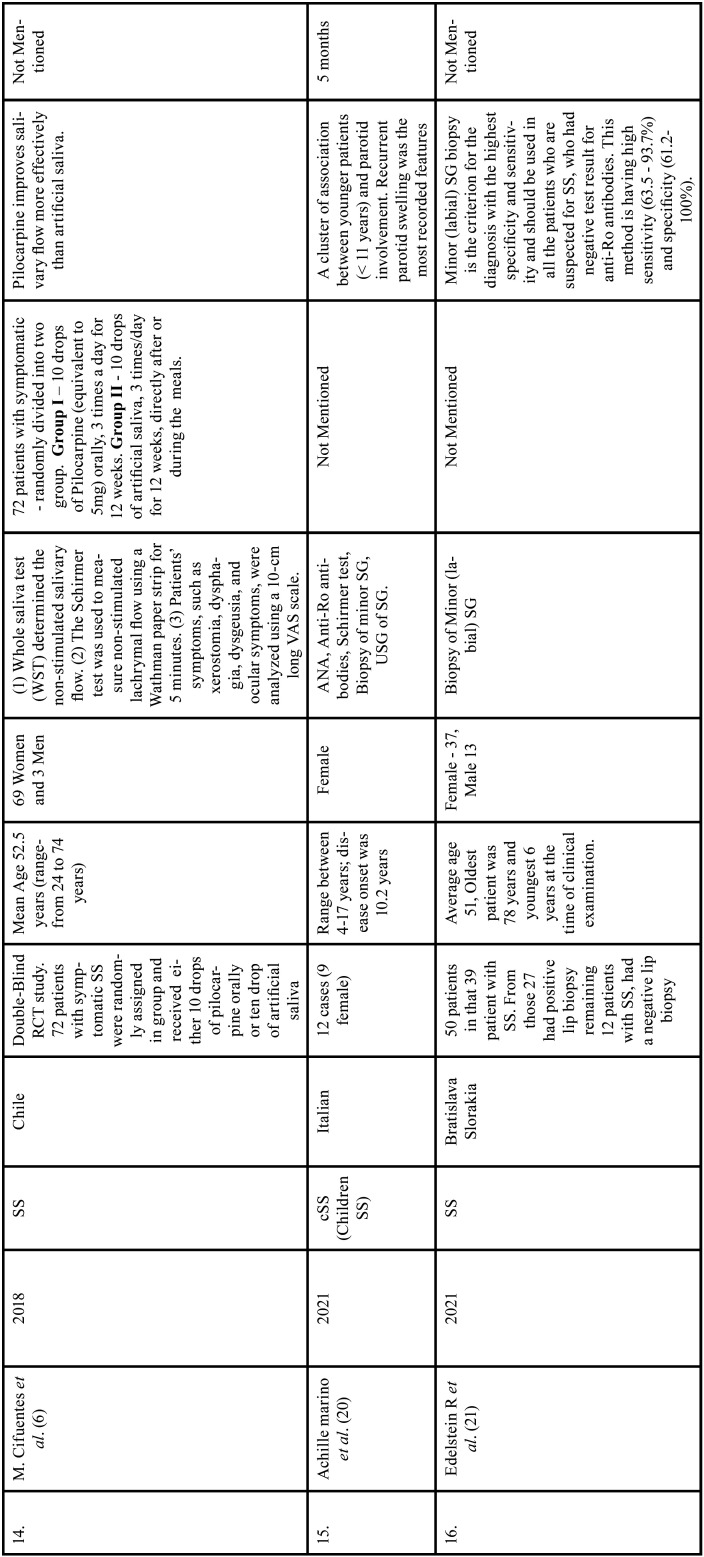
Charaterstics of included studies based on Population, Intervention, Comparison, and Outcome(PICO) model.

● Prednisolone

Marcosde Mendonca Invernici *et al*. (2014), (14) reported a case of 58-year-old female diagnosed with SS. She started the use of corticosteroid prednisone, 20 mg/daily, in order to control inflammation and pain. Authors concluded that it was possible to treat partially edentulous patients with SS associated with RA and diabetes, using corticosteroids and oral hyperglycemic therapy with dental implants and fixed prosthesis. Patient’s comfort was significantly improved with this treatment and there was no bone loss reported even after 6 years.

Kaufman *et al*. (2008) (15) presented a rare case report of pSS with proteinuria and hypokalaemic tetraparesis due to sever interstitial nephritis, which was treated successfully with higher dosage of steroids (prednisone 20 mg/day), liquifilm eye drops and azathioprine. It was concluded that pSS patients with renal involvement can be treated with immunosuppressive therapy.

Catherine M *et al*. (2001) (16) reported a case report of African-American girl aged 14 years with bilateral parotid swelling and generalized tooth sensitivity, especially when drinking cold carbonated beverages. The patient was managed with systemic corticosteroids in low dose for the parotitis and the arthritis.

YO Ueda *et al*. (2014) (17) reported a case of 69-year-old female, diagnosed with pSS 23 years ago, developed interstitial cystitis [IC]was successfully treated with tacrolimus and prednisolone combination therapy.

● Biologic response modifier vs placebo

MARTIN J. CUMMINS *et al*. (2003) (18) reported a clinical trial of 2 phase III in pSS patients which included 497 subjects who received 150 IU of human IFN-α or matching placebo 3 times/day by the oral route for 24 weeks. IFN-α administered by the oral route at a lower dosage can lead to remarkable increase in UWS flow in the patients with pSS, without causing and significant adverse events.

Adverse events

Patients administered with the pilocarpine and cevimeline were reported with the adverse events like sweating, nausea, headache, palpitations respectively. No adverse events association was noted with salivary electrostimulation. Infections, serum sickness and infusion reactions were also observed in patients taking rituximab. Adverse effects on gastro-intestinal system were found to be linked with IFN-α.

## Conclusions

Analysis of studies showed favorable effects of pilocarpine, rituximab and IFN-α in the reduction of dry mouth symptoms. The use of other treatment strategies such as low-level LASER therapy, cognitive behavioral therapy, electrostimulation, etc. cannot be generalized based on the current evidence. Studies based on cognitive behavioral therapy should be carried out for SS patients as this therapy has been documented to have potential to improve QoL of patients. Effectiveness of therapies such as hydroxychloroquine, immunosuppressants, hydroxychloroquine, infliximab should be validated by further RCTs in SS patients.
